# Impact of Genetic Counseling and Connexin-26 and Connexin-30 Testing on Deaf Identity and Comprehension of Genetic Test Results in a Sample of Deaf Adults: A Prospective, Longitudinal Study

**DOI:** 10.1371/journal.pone.0111512

**Published:** 2014-11-06

**Authors:** Christina G. S. Palmer, Patrick Boudreault, Erin E. Baldwin, Janet S. Sinsheimer

**Affiliations:** 1 Department of Psychiatry & Biobehavioral Sciences, University of California Los Angeles, Los Angeles, California, United States of America; 2 Department of Human Genetics, University of California Los Angeles, Los Angeles, California, United States of America; 3 Institute for Society and Genetics, University of California Los Angeles, Los Angeles, California, United States of America; 4 Department of Deaf Studies, California State University Northridge, Northridge, California, United States of America; 5 Departments of Biomathematics and Biostatistics, University of California Los Angeles, Los Angeles, California, United States of America; Centro de Investigación Príncipe Felipe – CIPF, Spain

## Abstract

Using a prospective, longitudinal study design, this paper addresses the impact of genetic counseling and testing for deafness on deaf adults and the Deaf community. This study specifically evaluated the effect of genetic counseling and Connexin-26 and Connexin-30 genetic test results on participants' deaf identity and understanding of their genetic test results. Connexin-26 and Connexin-30 genetic testing was offered to participants in the context of linguistically and culturally appropriate genetic counseling. Questionnaire data collected from 209 deaf adults at four time points (baseline, immediately following pre-test genetic counseling, 1-month following genetic test result disclosure, and 6-months after result disclosure) were analyzed. Four deaf identity orientations (hearing, marginal, immersion, bicultural) were evaluated using subscales of the Deaf Identity Development Scale-Revised. We found evidence that participants understood their specific genetic test results following genetic counseling, but found no evidence of change in deaf identity based on genetic counseling or their genetic test results. This study demonstrated that culturally and linguistically appropriate genetic counseling can improve deaf clients' understanding of genetic test results, and the formation of deaf identity was not directly related to genetic counseling or Connexin-26 and Connexin-30 genetic test results.

## Introduction

Connexin-related deafness is the most common worldwide form of hereditary deafness [1]. Furthermore, the relative frequency of Connexin-related deafness has increased several-fold over the last 100 years in the United States in the non-Hispanic white population as a result of marriages based on linguistic homogamy (signed language) [2], [3]. Thus, deafness-causing variants in the genes underlying Connexin-related deafness (*GJB2* and *GJB6*, also sometimes referred to as Connexin-26 and Connexin-30, respectively) appear to play a significant role in the history and formation of the Deaf community and Deaf culture in the United States. In this article, the term Deaf (with a capital D) is used to refer to individuals who are members of the Deaf community, a distinct cultural group. The term Deaf is distinct from deaf (with a lower case d), which refers simply to an audiologic phenotype; individuals within the Deaf community may be deaf or hard-of-hearing [4]. Accordingly, Deaf identity and Deaf community (uppercase D) are used as Deaf culture descriptors only, and deaf identity and deaf community (lowercase d) are used when a collective term is needed to describe generic identity and community including Deaf and hearing affiliations.

The Deaf community has a distinct culture with its own beliefs, customs, attitudes, language (i.e., American Sign Language [ASL] in the United States), and behavioral norms. This culturally collective group views being deaf as a trait or characteristic of human diversity that is important to their culture [5]. Since the identification of deafness-causing variants in these two genes over a decade ago, a number of studies have demonstrated that some deaf individuals are interested in genetic testing to learn why they are deaf [6]–[9]. Although deafness-causing variants in Connexin-26 and Connexin-30 may be important for the Deaf community, the availability of deaf genetic testing raises a variety of issues for the Deaf community.

One important issue to consider is whether or not *knowing* genetic information challenges or changes an individual's deaf identity. Deaf identity is an individual's self-identification with deafness, which relates directly to and is influenced by the individual's identification with the Deaf community. Research suggests that individual differences yield four different deaf identity orientations: hearing, marginal, Deaf (also called immersion), and bicultural (sometimes referred to as equal involvement in deaf and hearing communities) [10]–[15]. Within this framework individuals hold a hearing identity when the mainstream hearing society is their primary frame of reference. These individuals identify with the hearing society in attitude, behavior, and communication style, and they tend to view being deaf from a medical-pathological perspective. Individuals with marginal identity are ambivalent about their deafness and their cultural frame of reference (hearing, deaf or hard-of-hearing). Individuals who are heavily engaged with the Deaf community, feel a strong sense of “Deaf” pride, view deafness as a personal characteristic, and communicate with sign language, are considered to have an immersion or culturally Deaf identity. Finally, individuals with a bicultural identity feel comfortable with both deaf and hearing people, appreciate both cultures, and may be bilingual in sign and written or spoken languages. Variation in deaf identity (and hence cultural/community affiliations) may exist in part because most deaf individuals are not born into the Deaf community. Instead factors such as their language preference, educational experiences, and personal and social experiences play a large role in shaping their identity and affiliation with the both the Deaf and hearing communities [12], [16], [17].

Hence, the deaf community is composed of individuals who differ in their identity as a deaf person, that is, their cultural affiliation [18], [19]. However, studies have found that identities can shift and change as a result of a variety of factors, such as demographic (e.g., age), psychological, or contextual variables [20]–[22]. Deaf identity is no different. Deaf identity is complex and dynamic because the development of an individual's deaf identity is a process that evolves over time, is influenced by many factors including interactions with deaf and hearing peers [23]–[25], and may be context-dependent or in flux [18]. Dimensional measures such as the Deaf Identity Development Scale – Revised [13] and the Deaf Acculturation Scale [11], [26], [27] have been developed to provide more nuanced understanding of deaf identity rather than relying on a categorical assignment.

Research has shown that the extent to which one affiliates with the Deaf culture/community affects attitudes toward genetic testing in hypothetical situations where no testing has been offered [28]–[32] as well as situations where actual testing is offered [6]. Not only may deaf identity affect interest, knowledge, and attitudes toward genetic information, but deaf identity may also be *affected* by genetic information provided through genetic counseling and genetic testing. Changes in deaf identity are one possible route through which the individual impact of genetic testing can affect the larger Deaf community. Sufficient concern has been raised about susceptibility to changes in deaf identity that may affect survival and growth of the Deaf community that there are studies examining effects of deaf-related technologies on deaf identity. For example, to address concern about the effects of cochlear implant use on the Deaf community/culture, several studies have examined deaf identities in cochlear implant (CI) users and non-users [33], [34]. Both studies found that CI users more frequently endorsed items measuring a hearing identity compared to those without a CI. However, one study also found that CI users endorsed beliefs about deafness and Deaf culture that were similar to non-users [34], and the other study found similar number of participants with bicultural identities in both groups [33]. These results may play a role in allaying potential concerns about the impact of this particular technology on the Deaf community.

Deaf culture and the Deaf community cannot be sustained without a clear sense of identity and belonging. Changes in deaf identity are one possible route through which the individual impact of genetic testing can affect the Deaf community. Thus, an important issue to consider is whether or not genetic information challenges or changes one's deaf identity. Examining the effect of deaf genetic testing on deaf identity is important because if all prelingually deaf individuals had genetic testing, about half will be found to have a genetic explanation for why they are deaf [35]. We previously demonstrated that deaf individuals who learned that they have Connexin-related deafness experienced psychological well-being and that those who received a negative or inconclusive genetic test result experienced some psychological distress [36]. Hence, there already is empirical evidence that deaf genetic information has an impact on deaf individuals. Moreover, there is some evidence outside of the realm of deaf identity that identity may be affected by genetic information, as genetic test results have been shown to influence self-concept [37] and self-esteem [38]. Thus, if the receipt of a *genetic explanation* for deafness has a different effect on deaf identity than the receipt of *no explanation* for deafness, there could be a profound impact on the Deaf community and Deaf culture.

Individuals often learn genetic test results from a genetic counselor or geneticist through a result disclosure genetic counseling session. Genetic information can be complex to explain and understand, and the challenges of understanding the meaning of Connexin-related genetic test results have been documented among hearing parents of a deaf child [39]. As an additional layer of complexity, the unique language and culture shared by many deaf and hard-of-hearing individuals challenge the norms for both acquisition and provision of genetics information which could impact the effectiveness of result disclosure genetic counseling sessions. ASL is the primary language for culturally Deaf individuals within the U.S. Deaf community, and English is usually their second language. However, some individuals may opt to use spoken English, a combination of ASL and English, a form of signed English, or, in some rare instances, a unique signed language developed and used within the home of the deaf individual. Importantly, the use of signed language interpreting during genetic counseling to convey information between parties using two different communication modalities introduces an additional layer of complexity to the understanding of genetic concepts [40], [41].

Studies specifically examining effectiveness of genetic counseling to enhance genetics knowledge outcomes in cultural and linguistic minority groups are beginning to emerge [42]–[44]. We recently demonstrated that deaf adults' genetics knowledge and understanding of the etiologic heterogeneity of deafness, Connexin-related deafness, inheritance, and genetic testing, are enhanced by *pre-test* genetic counseling provided in a culturally and linguistically sensitive manner [40]. Although studies have found that understanding genetic test results is challenging, particularly negative and inconclusive results, there are data on the positive effect of genetic counseling on enhancing understanding of specific genetic test results among hearing parents of a deaf child [45]. Currently there are no empirical data regarding the effects of genetic counseling on deaf individuals' understanding of their actual genetic test results. The dearth of data on deaf individuals' understanding of their genetic test results poses a barrier to the development of culturally and linguistically tailored counseling strategies and the provision of anticipatory guidance to deaf clients in general, and specifically to Deaf individuals.

This paper addresses two major aims with data collected from deaf individuals participating in a prospective, longitudinal genetic counseling and testing study. The first aim was to determine the effect of genetic counseling or Connexin-26 and Connexin-30 genetic test results on participants' deaf identity; despite having sufficient statistical power we found no evidence that the degree of change in deaf identity differs between individuals receiving a genetic explanation and individuals receiving negative genetic test results over the course of the study. The second aim was to evaluate participants' comprehension of their specific genetic test results; and we found strong evidence that participants understood their test results following result disclosure genetic counseling.

## Materials and Methods

### Research Design

The Deaf Genetics Project (DGP) is a prospective, longitudinal study to examine the impact of genetic counseling and genetic testing (Connexin-26 and Connexin-30) on deaf adults and the deaf community. A multi-institutional, multi-disciplinary team employed a collaborative research model that integrated the Deaf cultural perspective, the hearing cultural perspective, the academic cultural perspective, and the community service perspective into the research design and implementation of a genetic counseling and testing study. The research team included board-certified genetic counselors and certified project staff sign language interpreters. Details on the sample, recruitment strategies, study protocol, and research team have been previously published [6], [36], [40], [46] and are described briefly below.

### Sample and Study Protocol

Individuals at least 18 years old, with unexplained sensorineural deafness since an early age were eligible to participate. Recruitment primarily took place in the Los Angeles, San Francisco Bay, and Riverside areas of California.

Individuals determined to be initially eligible had an audiological evaluation to confirm the presence of sensorineural deafness to ensure that Connexin-26 and Connexin-30 genetic testing was offered only to individuals for whom it was potentially relevant, i.e., those with early-onset sensorineural deafness. The first questionnaire (called the baseline questionnaire) was completed immediately following the audiology session. Individuals with confirmed sensorineural deafness then met with a genetic counselor for an in-person pre-test genetic counseling session. The genetic counselor explained the remaining study protocol, and provided general information about the genetic epidemiology of deafness, Connexin-related deafness, and genetic testing. Participants interested in pursuing genetic testing provided family and personal medical history information and a buccal sample for genetic analysis. The buccal sample was sent to a University of California, Los Angeles (UCLA) clinical laboratory for genetic analysis. The second questionnaire (called the pre-test counseling questionnaire) was completed immediately following the pre-test genetic counseling session.

Participants returned for another in-person genetic counseling session when their genetic test results were available. The genetic counselor explained the genetic test results, put them in the context of the participant's family and medical history and etiologic heterogeneity of deafness, and answered participants' questions. Participants received a copy of their genetic test report and a genetic counseling summary letter. In some cases, participants received additional information about genetics clinics in their area, either because they were interested in continuing to try to learn why they are deaf, or because something of clinical importance with a strong genetic component was noted in the family history, e.g., early onset breast cancer. About one month and six months after participants received their genetic test results, they were asked to complete the third questionnaire (1-month post-test questionnaire) and the final questionnaire (6-month post-test questionnaire), respectively. The four questionnaires assessed nearly identical information thus allowing us to examine the effect of genetic counseling and genetic information in a longitudinal framework.

The study was approved by the California State University Northridge (protocol: Deaf Genetics Study) and UCLA (protocol: 06-05-065/10-001193) institutional review boards. All research personnel obtained training on Health Insurance Portability and Accountability Act privacy rules, which provided additional protection to participants when a third party (i.e., an interpreter) was present during genetic counseling sessions. Genetic counseling and genetic testing were provided at no charge to participants in the study. All participants provided written informed consent, and as part of this process they were informed that they could pursue genetic counseling and genetic testing outside of this study on their own.

### Measures

The study questionnaires used standard as well as newly developed items to assess demographic factors, reasons for genetic testing, attitudes toward genetic testing, knowledge and understanding of genetics and genetic testing, cultural affiliation and deaf identity, and a variety of psychological and behavior measures. Questionnaires were translated into ASL using a translation-back translation procedure in which a bilingual ASL/English Deaf individual translated the original English version into ASL, another bilingual ASL/English individual back-translated the ASL version to English, and any inconsistencies in meaning were identified and resolved [47], [48]. Questionnaires were available to participants in English text (paper and online versions), ASL (online video streaming), and an online dual language format of both English text and ASL video.

#### Deaf Identity

Deaf identity was assessed using the Deaf Identity Development Scale-Revised (DIDR-R) [13]. This 47-item instrument contains 4 subscales (hearing, marginal, immersion, bicultural). Each subscale is comprised of 10–13 items evaluated on a 5-point Likert scale of strongly agree to strongly disagree. Investigations of the DIDS-R demonstrate that four distinct categories can be measured [10], [13]; that the subscales have good internal reliability with Cronbach's alpha ranging from 0.78–0.87; that the direction of correlations between subscales are generally consistent with the theory that deaf identity follows a developmental process [10], [13]; and that there are differences in mean subscale scores between students attending Gallaudet University compared to members of the Association for Late-Deafened Adults-Boston [10], and among hard of hearing individuals, prelingually deaf individuals, and postlingually deaf individuals [13]. An average subscale score was computed for each subject at each assessment time point by summing the individual subscale items and dividing by the total number of items in the subscale. This yielded average scores ranging from 1 (strongly agree) to 5 (strongly disagree) for each subscale and each time point.

#### Understanding Specific Genetic Test Result

Participants' understanding of their specific test result was assessed with two items: (1) *perceived chance of having Connexin-related deafness* was assessed by asking them to judge the likelihood that they have Connexin-related deafness on a 4-point Likert scale ranging from ‘not at all likely’ to ‘definitely’; and (2) *belief about why deaf* was assessed by asking participants to select the *most likely* reason they are deaf. At baseline, respondents were presented with 6 options: “It is genetic, because other people in my family are deaf/hard-of-hearing”; “It is genetic, even though no one else in the family is deaf/hard-of-hearing”; “Something happened when my mother was pregnant with me”; “Something happened while my mother was giving birth to me”; “Something happened to me after I was born”; and “It is unknown.” The first two options were then classified as “genetic,” the next three options were classified as “non-genetic” and the last option was classified as “unknown” for subsequent analyses. At each of the other three timepoints, participants selected from the following options: “genetic,” “not genetic,” “has not been determined”.

#### Connexin Group Classification

Participants were classified into one of three Connexin result groups: Connexin-positive, Connexin-negative, Connexin-inconclusive. Individuals were classified as Connexin-positive if their genetic test result clearly explained why they are deaf, i.e., they have two known Connexin-26 or Connexin-30 deafness-causing variants. Individuals were classified as Connexin-negative if their genetic test result did not identify any Connexin-26- or Connexin-30-related deafness variants. Individuals were classified as Connexin-inconclusive if their genetic test result did not provide enough information to determine if they have Connexin-related deafness (i.e., only one Connexin-related deafness variant was identified). Preliminary analyses suggested that some results were substantively changed by combining the Connexin-negative and Connexin-inconclusive groups, hence analyses do not combine these two groups.

#### Demographic Characteristics

Age, sex, ethnicity/race, high school program, income, highest level of education achieved, cultural affiliation, linguistic preference during interactions with hearing audiology and genetic counseling project staff, and family history of deaf relatives were assessed at baseline. For consistency, these variables are treated using the same categorizations described in other publications from this study [6], [36], [40]. Potential confounders such as enrollment site, genetic counselor, and interpreter were recorded for each participant.

## Analyses

Data from participants who completed at least one of the two post-test questionnaires are analyzed. Within that pool, responses were missing for 0.45% of the DIDS-R subscale items across the four assessment time points. To maximize the sample size, we used simple imputation to fill in missing data on individual DIDS-R subscale items, with the exception that DIDS-R subscale scores were not computed for one subject per time point because of substantial missing data.

Descriptive statistics were produced and reviewed for the presence of outliers and data errors. Bivariate analyses are performed using Fisher's exact test and ANOVA. For the primary analyses, we performed repeated measures regression analyses to determine the effect of genetic counseling or genetic test results on participants' deaf identity and their perceived chance of having Connexin-related deafness. For these analyses the interaction between Connexin group and assessment time point was the primary predictor variable in a model that included the corresponding separate main effects of Connexin group and time point. Age, education, language preference, family history of deaf relatives, and high school program were included as covariates because they are significantly associated with cultural affiliation in this sample [6]. Age was treated as a dichotomous variable as either above or below the sample median age of 45.5 years. In the case of a significant interaction term, post-hoc two-way comparisons were conducted to identify specific group differences using Tukey's HSD test [49], which controls for Type I experiment-wise error rate. Secondary or subset analyses were also performed as additional checks on participants' understanding of the genetic test results. Analyses were conducted using SAS version 9.3 [50]. Statistical significance was set at α = 0.05.

Analyses that include language preference exclude individuals who were categorized in the “other” language group as there were too few (*n* = 2) in this group for meaningful comparison. The number of individuals self-reported as non-white was relatively small (*n* = 40) and consisted of a number of ethnic groups, prohibiting using ethnicity/race as a covariate. Instead, to address the robustness of the results, we re-ran the repeated measures regression analyses with the subset of participants who marked white as their ethnic/racial category. These analyses yielded similar parameter estimates, providing evidence that the results from the larger sample are robust with respect to race/ethnic heritage.

For the repeated measures analysis of deaf identity, we conducted power analyses using PASS 11's mixed model procedure to determine the minimally detectable interaction between Connexin group and assessment time point for each DIDS-R subscale with 80% power and a two-sided significance level of 0.05. Because the Connexin inconclusive group was small and it is difficult to hypothesize the direction of change in values for this group, we limited our power assessments to compare two levels of the factor Connexin, positive and negative, in order to determine the minimum detectable group by time interaction. The mixed model power repeated measures procedure relies on simulations; with each power analysis conducted we used 500 simulation iterates providing us with precision of +/−2%. We used the same covariance structures that were used in actual data analyses and all parameter estimates, except the interaction effect sizes, were fixed to those observed values from the actual data analyses.

## Results

### Sample Demographics

**Figure 1:**
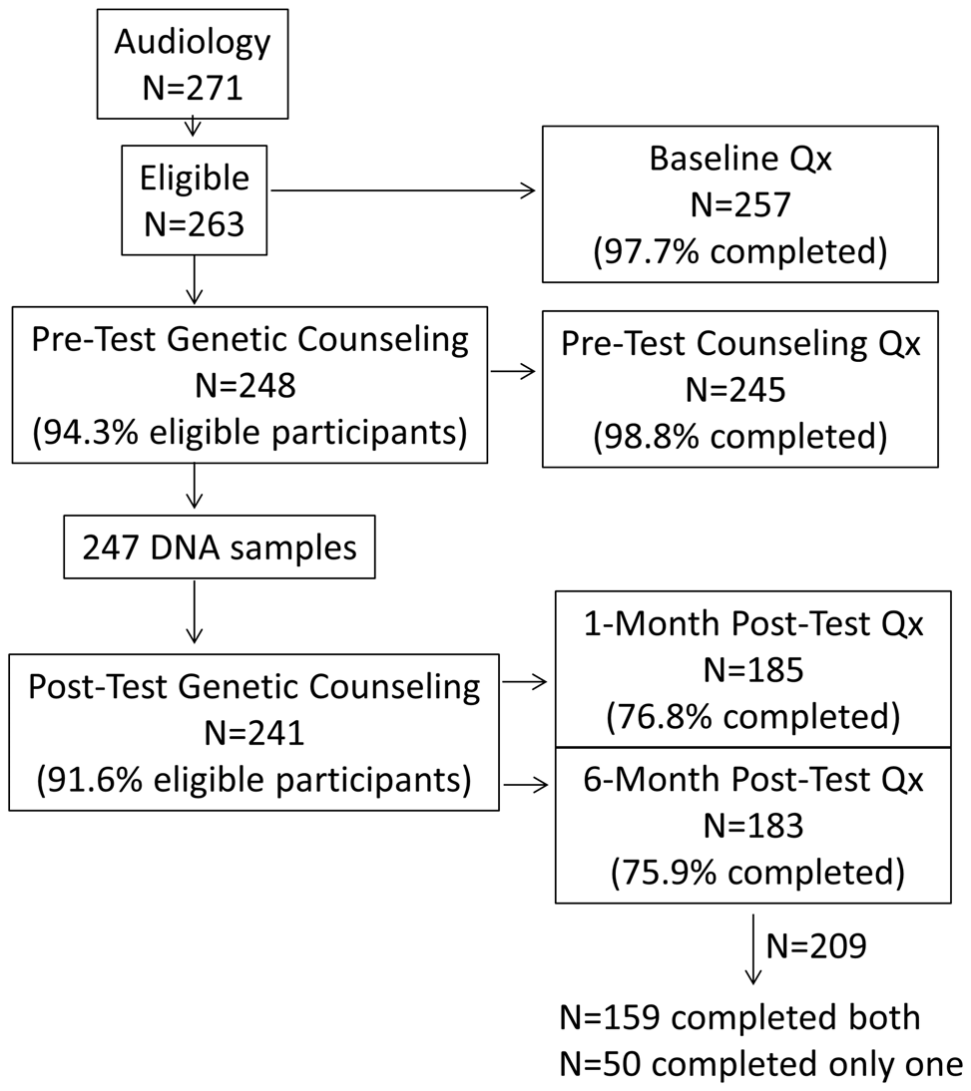
Study sample and questionnaire completion rates. Qx = questionnaire.

A total of 271 participants completed the audiology evaluation portion of the study, of which 263 were determined to be eligible to participate in the genetic counseling and testing part of the study. Figure 1 depicts the sample size and questionnaire completion rates at each step in the research protocol.

Among the 263 eligible individuals, 241 (91.6%) continued through the protocol to receive their genetic test results; 22 (8.4%) did not. Of those 22 subjects, 15 stopped participation after audiology evaluation (3 subjects withdrew after the audiology evaluation and all collected data on these individuals was destroyed; 3 declined to continue; 9 were not able to be scheduled for a pre-test genetic counseling session after at least one attempt), while the other 7 stopped participation after the pre-test genetic counseling session in which they provided a DNA sample for testing (3 declined to continue, 3 were not able to be scheduled for a test result disclosure session after at least one attempt, and 1 did not attend a scheduled test result disclosure session).

We compared subjects who received their genetic test results (*n* = 241) to those who ceased participation prior to that point (data available on *n* = 19) on the demographic variables evaluated in this study, DIDS-R subscale scores, enrollment site, genetic counselor, and interpreter. The results demonstrated that those who ceased participation were more likely to be male (*p* = 0.001) and to use ASL (*p* = 0.03), were less likely to self-identify as non-Hispanic white (*p* = 0.01), and more strongly endorsed the DIDS-R immersion subscale items (*p* = 0.004) than those who continued through the protocol to receive their genetic test results. About a quarter (26.2%) of participants were enrolled in the last six months of the study, and individuals who ceased participation were disproportionately represented in this group (63.1%) (*p* = 0.0005).

Among the 241 subjects who received their genetic test results, 100% completed the baseline and pre-test counseling questionnaires. Among these 241 subjects, 209 completed at least one post-test questionnaire, with the following breakdown: 159 completed both post-test questionnaires (complete responders), 50 completed only one post-test questionnaire (partial responders), and 32 (13.3%) did not complete any post-test questionnaires (non-responders). One individual provided unusable questionnaire data and was excluded from all analyses. Comparisons of complete, partial, and non-responders did not reveal statistically significant differences on any of the demographic variables evaluated in this study, on DIDS-R subscale scores, in the distribution of Connexin test results, or as a function of enrollment site, genetic counselor, interpreter, or length of time between providing a DNA sample and Connexin result disclosure (*p*'s>0.05), thus allowing us to treat the data as missing at random. Cronbach's α was computed from our sample for the DIDS-R subscales and found to be acceptable at each of the four time points (hearing subscale: α's ranged from 0.85–0.87, marginal subscale: α's ranged from 0.88–0.91, immersion subscale: α's ranged from 0.80–0.84, bicultural subscale: α's ranged from 0.77–0.84).

Responses from the 209 subjects who completed at least one post-test questionnaire are the focus of our analyses, and demographic characteristics of this sample are provided in Table 1 by Connexin result category. In this study sample, 39.2% were classified as Connexin-positive (*n* = 82), 13.4% as Connexin-inconclusive (*n* = 28), and 47.4% as Connexin-negative (*n* = 99). The Connexin groups were compared on the demographic variables in Table 1 and found to be comparable on all but preferred language (*p* = 0.03) and family history (*p*<0.0001). Not surprisingly, the three Connexin groups differed in terms of the presence of deaf relatives where 75.6% of those in the Connexin-positive group had at least one closely related deaf relative compared to 57.1% and 37.4% of those in the Connexin-inconclusive and Connexin-negative groups, respectively. Furthermore, 73.2% of those in the Connexin-positive group preferred to use ASL with an interpreter with the genetic counselor, compared to 67.9% and 53.5% in the Connexin-inconclusive and Connexin-negative groups, respectively.

**Table 1:** Sample demographics by Connexin result.

		Connexin result			*p*
		Positive	Inconclusive	Negative	
Sample size		82	28	99	
Average age (SD), years		44.6 (15.9)	48.0 (17.4)	46.3 (15.3)	0.58
Female, % (n)		54.9 (45)	67.9 (19)	66.7 (66)	0.23
Ethnicity/race, % (n)	non-Hispanic white	85.4 (70)	82.1 (23)	76.8 (76)	0.34^a^
	Hispanic	7.3 (6)	3.6 (1)	12.1 (12)	
	Asian	4.9 (4)	14.3 (4)	9.1 (9)	
	Other	2.4 (2)	0	2.0 (2)	
High school program^b^, % (n)	Deaf	42.5 (34)	28.6 (8)	28.4 (27)	0.31
	Hearing	23.8 (19)	32.1 (9)	38.9 (37)	
	Mainstream	25.0 (20)	25.0 (7)	21.1 (20)	
	Mixed	8.8 (7)	14.3 (4)	11.6 (11)	
Median income, thousands of $		35–50	50–65	35–50	0.27
≥Bachelor degree, % (n)		57.3 (47)	53.6 (15)	56.1 (55)	0.93
Cultural affiliation, % (n)	Deaf community	63.4 (52)	53.6 (15)	48.5 (47)	0.20^c^
	Hearing community	6.1 (5)	14.3 (4)	7.2 (7)	
	Both communities	29.3 (24)	32.1 (9)	42.3 (41)	
	Neither community	1.2 (1)	0	2.1 (2)	
Language, % (n)	ASL, interpreter present	73.2 (60)	67.9 (19)	53.5 (53)	0.03^d^
	ASL and English, interpreter present	19.5 (16)	14.3 (4)	29.3 (29)	
	English, no interpreter present	6.1 (5)	17.9 (5)	16.2 (16)	
	Other	1.2 (1)	0	1.0 (1)	
Deaf 1^st^ - or 2^nd^ -degree relatives, % (n)		75.6 (62)	57.1 (16)	37.4 (37)	<0.0001

*Note.* ^a^Compares non-Hispanic white group to all other ethnic groups;

b As in Boudreault et al. 2010, deaf-based high school indicates predominantly ASL or coded communication in the classroom; hearing-based high school indicates predominantly oral instruction in the classroom without interpreter/support services; mainstream high school indicates public school that predominantly provides sign instruction with interpreter/support services; and mixed indicates attending two or more of the previously described high school programs;

c Individuals in “Neither Community” excluded from all analyses involving this variable due to small sample size;

d Individuals in “Other” indicated signed English or Pidgin Signed English and are excluded from analyses involving this variable due to small sample size.

### Deaf Identity

Prior to investigating the effect of genetic counseling or Connexin test results on participants' deaf identity we performed ANOVA to determine if change in DIDS-R subscale scores from pre-test (prior to learning the Connexin results) to 1-month post-test (after learning the Connexin results) was associated with a specific genetic counselor or sign language interpreter. No association was found between genetic counselor and any of the four DIDS-R subscale difference scores (*p*'s>0.05). There was a significant association between sign language interpreter and change on immersion subscale scores (*p* = 0.03); however, results were not substantively altered when interpreter was included as a covariate in the regression analysis described below, therefore genetic counselor and interpreter are not considered further in this subsection.

**Figure 2:**
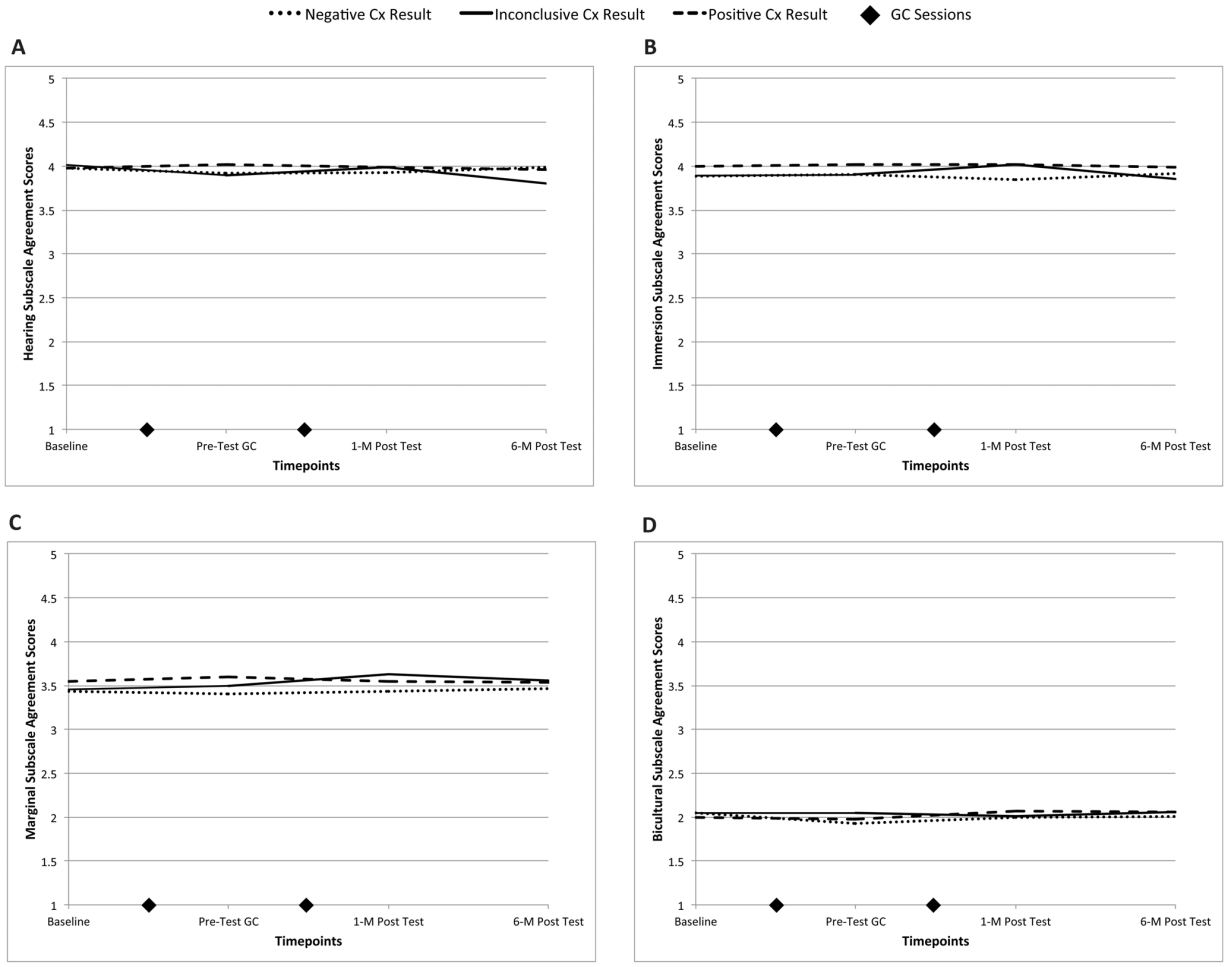
2A–2D: DIDS-R subscale agreement score least squares estimates by Connexin result group before and after genetic testing. A. hearing subscale, main effects: Connexin *p* = 0.84, time *p* = 0.08; interaction: Connexin x time *p* = 0.02. Non-significant pairwise comparisons at α = 0.05 at each timepoint. B. marginal subscale, main effects: Connexin *p* = 0.45, time *p* = 0.61; interaction: Connexin x time *p* = 0.25. C. immersion subscale, main effects: Connexin *p* = 0.32, time *p* = 0.32; interaction: Connexin x time *p* = 0.20. D. bicultural subscale, main effects: Connexin *p* = 0.88, time *p* = 0.23; interaction: Connexin x time *p* = 0.27. 1 = strongly agree, 5 = strongly disagree. Cx = connexin; GC = genetic counseling; M = month.

Repeated measures regression analysis with covariates was then performed to determine if participants' responses to these deaf identity subscales changed over the course of the study, particularly after they learned their genetic test results. Figures 2A–2D plot participants' least square estimates of the DIDS-R subscale scores by Connexin result group before and after receiving their genetic test results; and Table S1 provides the least square estimates and standard errors for the main effects, covariates, and interaction term. Regardless of Connexin result, endorsement of the bicultural subscale was strongest, and endorsement of the hearing and marginal subscales was weakest, at each time point.

#### Hearing Subscale (Figure 2A)

While controlling for the main effects of Connexin result (*F*_(2,189)_ = 0.17, *p* = 0.84) and time (*F*_(2,538)_ = 2.29, *p* = 0.08), we found that the interaction between Connexin and time was significantly associated with hearing subscale scores (*F*_(6,538)_ = 2.44, *p* = 0.02). The covariates preferred language (*F*_(2,189)_ = 9.78, *p*<0.0001), age (*F*_(1,189)_ = 7.59, *p* = 0.006), education (*F*_(1,189)_ = 31.78, *p*<0.0001), and family history (*F*_(1,189)_ = 6.18, *p* = 0.01) also were significantly associated with these scores. Individuals expressing stronger endorsement of this subscale tended to be English-users, older than 45.5 years, not have college degrees, and have no first or second degree deaf relatives. Although the Connexin and time interaction term was statistically significant, post-hoc pairwise analyses did not demonstrate significant group differences at α = 0.05 at any of the four timepoints.

#### Marginal Subscale (Figure 2B)

While controlling for the main effects of Connexin result (*F*_(2,189)_ = 0.81, *p* = 0.45) and time (*F*_(3,538)_ = 0.61, *p* = 0.61), we found no evidence that responses to marginal subscale items changed by genetic test result category before or after learning results [non-significant interaction between Connexin and time (*F*_(6,538)_ = 1.31, *p* = 0.25)]. However, the main effects of education (*F*_(1,189)_ = 38.59, *p*<0.0001) and family history (*F*_(1,189)_ = 10.50, *p* = 0.001) were significantly associated with marginal subscale scores averaged over the four assessment time points. On average an individual without a college degree and without deaf relatives more strongly endorsed items on the marginal subscale than an individual with a college degree and deaf family members. Age (*F*_(1,189)_ = 1.25, *p* = 0.26), language (*F*_(2,189)_ = 0.15, *p* = 0.85), and high school program (*F*_(3,189)_ = 2.48, *p* = 0.06) were not significant predictors of marginal subscale scores.

#### Immersion Subscale (Figure 2C)

While controlling for the main effects of Connexin result (*F*_(2,189)_ = 1.14, *p* = 0.32) and time (*F*_(3,538)_ = 1.16, *p* = 0.32), we found no evidence that responses to immersion subscale items changed by genetic test result category before or after learning results [non-significant interaction between Connexin and time (*F*_(6,538)_ = 1.43, *p* = 0.20)]. However, the main effects of preferred language (*F*_(2,189)_ = 32.90, *p*<0.0001) and high school program (*F*_(3,189)_ = 4.23, *p* = 0.006) were significantly associated with immersion subscale scores averaged over the four assessment time points. ASL-users and individuals who attended a deaf-based high school more strongly endorsed items on the immersion subscale than English-users or those who attended a hearing-based high school. Age (*F*_(1,189)_ = 0.61, *p* = 0.44), education (*F*_(1,189)_ = 0.46, *p* = 0.50), and family history (*F*_(1,189)_ = 0.24, *p* = 0.63) were not significant predictors of immersion subscale scores.

#### Bicultural Subscale (Figure 2D)

While controlling for the main effects of Connexin result (*F*_(2,189)_ = 0.13, *p* = 0.88) and time (*F*_(3,538)_ = 1.43, *p* = 0.23), we found no evidence that responses to immersion subscale items changed by genetic test result category before or after learning results [non-significant interaction between Connexin and time (*F*_(6,538)_ = 1.27, *p* = 0.27)]. However, the covariate preferred language (*F*_(2,189)_ = 20.36, *p*<0.0001) was significantly associated with bicultural subscale scores averaged over the four assessment time points. Individuals who use both ASL and English or predominantly ASL more strongly endorsed items on the bicultural subscale than English-users. Age (*F*_(1,189)_ = 0.09, *p* = 0.76), education (*F*_(1,189)_ = 0.54, *p* = 0.46), family history (*F*_(1,189)_ = 1.35, *p* = 0.25), and high school program (*F*_(3,189)_ = 1.41, *p* = 0.24) were not significant predictors of bicultural subscale scores.

#### Power Analysis

Power analyses demonstrate that our sample size is sufficient to detect moderate group by time interactions within the four subscales. Specifically, there is at least 80% power to detect an interaction between Connexin group and time that would result in an individual with a Connexin negative result experiencing on average a 0.14 unit greater shift towards *endorsement* of a hearing identity during their participation in the study than an individual receiving a Connexin-positive result would experience in the same amount of time. That is, as an example, after adjustment for the covariates, we could expect the change for a Connexin-negative individual to be −0.10 units (increased endorsement of the hearing identity) and the change for a Connexin-positive individual to be 0.04 units (increased rejection of the hearing identity). Similarly, there is at least 80% power to detect an interaction between Connexin group and time that would result in an individual with a Connexin-negative result experiencing on average a 0.14 unit greater shift towards *endorsement* of a marginal identity during their participation in the study than an individual receiving a Connexin-positive would experience in the same amount of time. Reflecting the anticipated direction of change, there is at least 80% power to detect an interaction between Connexin group and time that would result in an individual with a Connexin-negative result experiencing on average a 0.16 unit greater shift towards *rejection* of an immersion identity than an individual receiving a Connexin-positive would experience in the same amount of time. As an example, after adjustment for the covariates, we would expect the change for a Connexin-negative individual to be 0.11 units (a decrease in endorsement of an immersion identity) and the change for a Connexin-positive individual to be -0.05 units (a slight increase in endorsement of an immersion identity). Finally there is at least 80% power to detect an interaction between Connexin group and time that would result in an individual with a Connexin-negative result experiencing on average a 0.12 unit greater shift towards *rejection* of a bicultural identity during their participation in the study than an individual receiving a Connexin-positive would experience in the same amount of time.

Visual inspection of Figures 2A–2D supports the conclusions of the statistical analyses that the subscale scores are essentially unchanged over the four assessment time points. Unsolicited comments provided by participants at the end of the post-test questionnaires further bolster the conclusion that participants' deaf identity was not changed as a result of genetic testing.

“Thank you for doing this project. It *helped me re-affirm my view of myself as a proud Deaf person*.” – Participant with Connexin-positive result (italics added for emphasis)

“I decided to participate in the genetics test to see why I am Deaf. My family and I had a hunch that it was genetic, but this helped proved that and *really had no immediate effect on my life*. Thanks for doing this!” Participant with Connexin-positive result (italics added for emphasis)

“I didnt [sic] know what to think when I got the test results. Whether to be exhilarated, excited, upset or confused. *I just felt sort quiet*.” Participant with Connexin-negative result (italics added for emphasis)

#### Cultural Affiliation and Number of Connexin Deafness-Causing Variants

Although these analyses provide support that Connexin result does not appear to *change* deaf identity in this sample of deaf adults, we hypothesized that culturally Deaf individuals would be more likely to be Connexin-positive, i.e., that cultural affiliation is associated with Connexin result. This hypothesis is based on evidence that the frequency of Connexin-related deafness has increased several-fold over the last 100 years in the US in the non-Hispanic white population as a result of marriages based on linguistic homogamy (signed language) [2] which is important for Deaf culture. Given the observed stability of the DIDS-R subscale scores over time in this sample, we categorized participants by their self-reported cultural affiliation at baseline to test this hypothesis. This categorical measure of cultural affiliation has been shown to correlate well with relevant variables such as preferred language, high school program, and involvement in the Deaf community, as well as responses to the DIDS-R subscales at baseline in this sample [6].

We found a significant correlation between participants' cultural affiliation and number of Connexin deafness-causing variants (FE *p* = 0.04), focusing on the non-Hispanic white subsample (*n* = 165) to avoid confounding with ethnic ancestry or potential cross-cultural differences. Specifically, 50.53% (48/95) of those who reported affiliation with the Deaf community had two Connexin deafness-causing variants (i.e., Connexin-related deafness) compared to 30.51% (18/59) of those who reported affiliation with both Deaf and hearing communities and 27.3% (3/11) of those who reported affiliation with the hearing community. When re-analyzed in the presence of language, age, education, family history, and high school program, the association between cultural affiliation and number of deafness-causing variants remained significant (*p* = 0.05). Overall these results suggest that the Connexin-26 and Connexin-30 genes are intimately connected to Deaf culture in the US, but individuals who have genetic counseling, Connexin testing, and learn their results do not experience a change in their deaf identity.

### Understanding Specific Connexin Result

#### Perceived Chance

We next assessed participants' understanding of their specific Connexin result by examining whether their responses to the *perceived chance* item “How likely is it that you have Connexin-related deafness?” was consistent with their actual Connexin test result, with the expectation that those who received a Connexin-positive result would be more likely to indicate that they have Connexin-related deafness than those who received a Connexin-inconclusive or Connexin-negative result. The majority of participants felt it was at least somewhat likely that they had Connexin–related deafness at baseline and immediately following the *pre-test* genetic counseling session (83.7%, 82.4%, respectively), but prior to learning their Connexin results. At 1- and 6- months after receiving the genetic test results, 74% of those who received a Connexin-positive result indicated that they *definitely* have Connexin-related deafness. In contrast, among those who received a Connexin-negative test result, 71.3% at 1-month and 68.6% at 6-months post-test result indicated that they felt it was *not at all likely* that they have Connexin-related deafness. The responses of the Connexin-inconclusive group are spread across all four response categories at 1-month and 6-months post-test result (not at all likely: 26.1%, 36.4%; somewhat likely: 43.5%, 27.3%; very likely: 17.4%, 22.7%; definitely: 13.0%, 13.6%), revealing the challenge of personalizing the meaning of an inconclusive test result.

**Figure 3:**
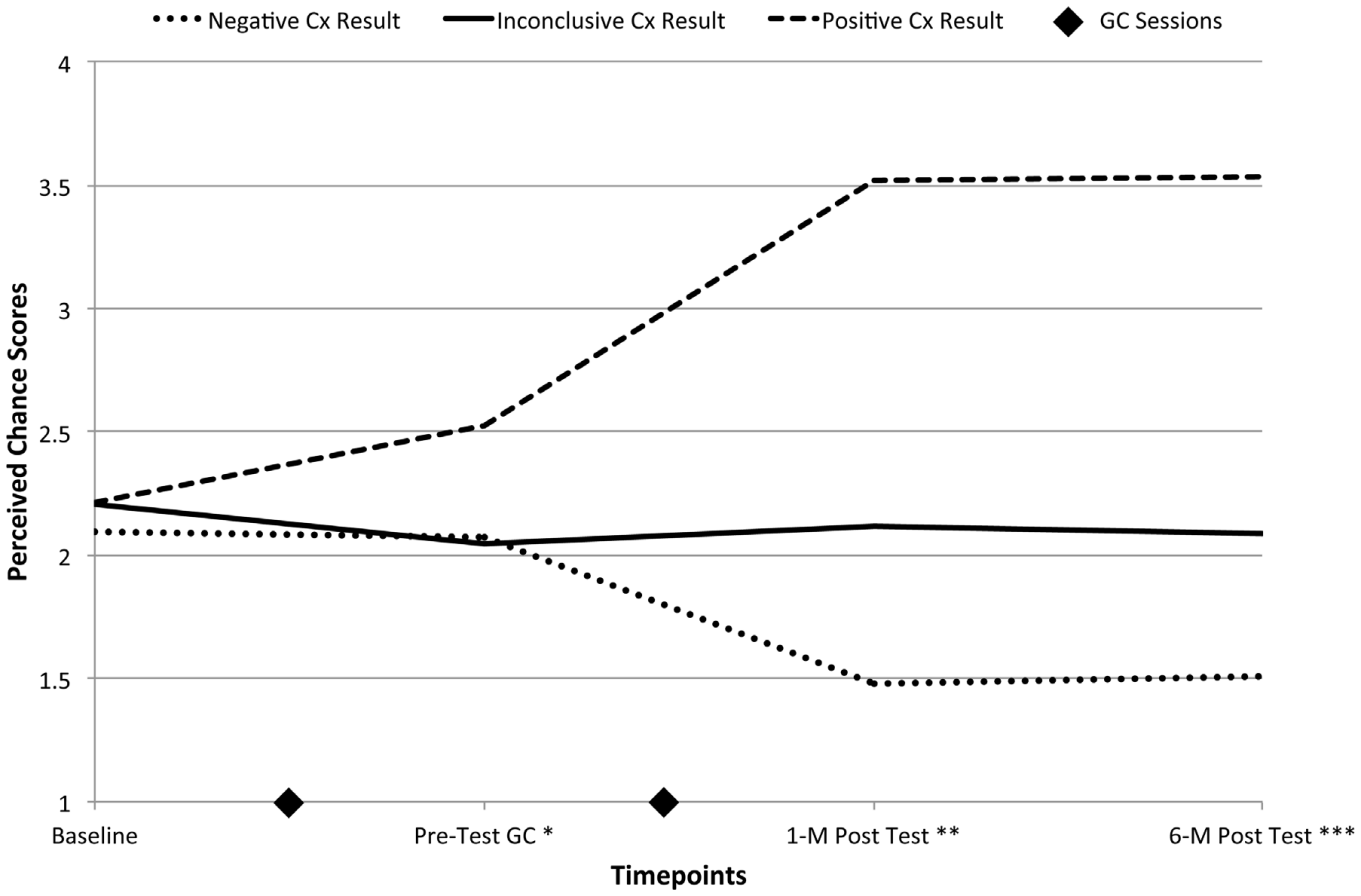
*Perceived chance* least squares estimates by Connexin result group before and after genetic testing. How likely is it that you have Connexin-26 related deafness? 1 = not at all likely, 2 = somewhat likely, 3 = highly likely, 4 = definitely. Main effects: Connexin *p*<0.0001, time *p* = 0.01; interaction Connexin x time *p*<0.0001; *Significant difference between Connexin-positive and Connexin-negative groups *p* = 0.02, and Connexin-positive and Connexin-inconclusive groups *p* = 0.02; **Significant difference between Connexin-positive and Connexin-negative groups *p*<0.0001, Connexin-positive and Connexin-inconclusive groups *p*<0.0001, and Connexin-negative and Connexin-inconclusive groups *p* = 0.0003; ***Significant difference between Connexin-positive and Connexin-negative groups *p*<0.0001, Connexin-positive and Connexin-inconclusive groups *p*<0.0001, and Connexin-negative and Connexin-inconclusive groups *p* = 0.0006. Cx = connexin; GC = genetic counseling; M = month.

Repeated measures regression analysis with covariates was then performed to determine if participants' responses to *perceived chance* changed over the course of the study, particularly after they learned their genetic test results. Figure 3 plots participants' least square estimates of *perceived chance* responses before and after receiving their genetic test results by Connexin result group; and Table S1 provides the least square estimates and standard errors for the main effects, covariates, and interaction term. The analysis demonstrated a statistically significant interaction between Connexin result and time (*F*_(6,530)_ = 50.26, *p*<0.0001) indicating that participants' perceived chance of having Connexin-related deafness changed by Connexin result category over the course of the study, while controlling for the main effects of Connexin result (*F*_(2,189)_ = 110.15, *p*<0.0001) and time (*F*_(3,530)_ = 3.65, *p* = 0.01), language preference (*F*_(2,189)_ = 0.79, *p* = 0.46), age (*F*_(1,189)_ = 0.25, *p* = 0.62), education (*F*_(1,189)_ = 0.38, *p* = 0.54), family history (*F*_(1,189)_ = 29.25, *p*<0.0001), and high school program (*F*_(3,189)_ = 5.14, *p* = 0.002). Posthoc pair-wise Tukey tests demonstrated that the perceived chance ratings of the three Connexin result groups did not differ at baseline, but significantly differed at the other three timepoints (Figure 3). Unexpectedly, the Connexin-positive group rated their chance of having Connexin-related deafness higher than the other two groups following pre-test genetic counseling. This finding could reflect that pre-test genetic counseling facilitated a good understanding of the meaning of participants' medical history and family history and set appropriate expectations for a participant's test result. The perceived chance ratings differed among all three groups at 1-month post-test and 6-month post-test. At both of these timepoints, the Connexin-positive group indicated a high level of certainty that they have Connexin-related deafness, the Connexin-negative group indicated a high level of certainty that they do not have Connexin-related deafness, and the Connexin-inconclusive group yielded an average score indicative that there remains some chance that they have Connexin-related deafness. These results provide evidence that participants' subjective assessments of Connexin-related deafness are consistent with the actual test results.

#### Connexin Result and Belief Why Deaf

**Figure 4:**
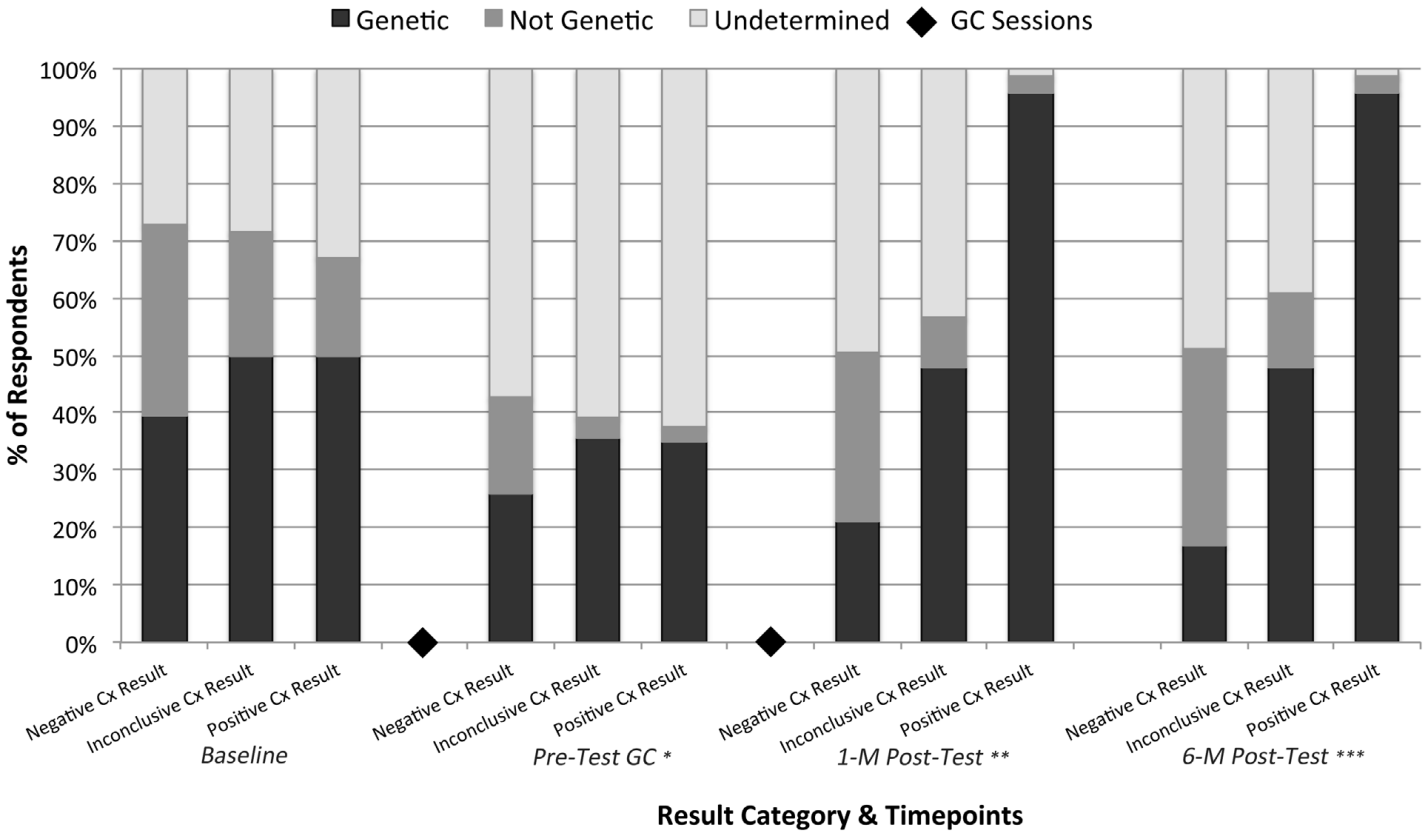
Responses to “belief about why deaf” by assessment timepoint. *p = 0.02; **p<0.0001; ***p<0.0001; Cx = connexin; GC = genetic counseling; M = month.

As a check on participants' understanding of their genetic test results, we performed two additional analyses. First, we evaluated the relationship between Connexin result and belief about why deaf, with the expectation that those with a Connexin-positive result would be more likely to report that they have a genetic form of deafness, and those with a Connexin-negative and Connexin-inconclusive result would be more likely to report that they are deaf for reasons ‘not determined’ after they learned their genetic test results. As shown in Figure 4, at baseline the distribution of participant responses for the reason they are deaf did not differ as a function of their underlying Connexin result (FE *p* = 0.16), and nearly a third reported that the reason they are deaf was undetermined. While the percentage of participants reporting an undetermined reason for their deafness increased to ∼60% at the pre-test genetic counseling assessment time point, prior to knowing their Connexin test result, those in the Connexin-negative group were less likely to indicate a genetic explanation compared to the Connexin-positive and inconclusive groups (FE *p* = 0.02). This result provides additional evidence that pre-test genetic counseling appropriately set expectations about genetic test results. One-month following receipt of Connexin results,>95% of participants with a Connexin-positive result reported a genetic explanation for their deafness; the Connexin-inconclusive group was fairly evenly split between attributing a genetic explanation and reporting that the etiology of their deafness was undetermined; and the Connexin-negative group was least likely to report that they have a genetic type of deafness (FE *p*<0.0001). Of note both the Connexin-negative and Connexin-inconclusive groups were more likely to report that the reason they are deaf is ‘undetermined’ than ‘not genetic’. This result suggests that communication between the genetic counselor and participant was effective in conveying the important concepts of genetic heterogeneity and that ruling out one genetic explanation does not imply ‘not genetic’. Results were similar at 6-months following receipt of Connexin results (FE *p*<0.0001).

Second, we examined the how participants' understood their Connexin result in light of their family history of deaf relatives. Although we demonstrated that those who received Connexin-negative results were less likely to report that they have Connexin-related deafness, it is clear that those with deaf relatives have more reason to believe they have a *genetic* form of deafness than those without deaf relatives. On the other hand, although those with Connexin-positive results were more likely to indicate that they have Connexin-related deafness, they also should believe that they have a genetic form of deafness *regardless* of their family history.

Irrespective of Connexin result category, it is clear at baseline that family history was associated with participants' beliefs about why they are deaf. Specifically, those with deaf relatives were more likely to attribute a genetic explanation to the reason they are deaf compared to those with no deaf relatives (Connexin-negative group, *p*<0.0001; Connexin-inconclusive group, *p*<0.0001; Connexin-positive group, *p* = 0.01). Following pre-test genetic counseling, this association continued to be statistically significant for the Connexin-negative and Connexin-inconclusive groups (*p*<0.0001, *p* = 0.02, respectively). Although the Connexin-positive group followed the same trend (40% of those with deaf relatives attributed a genetic explanation to their deafness compared to 20% of those without deaf relatives), the association was not statistically significant (*p* = 0.17). One-month following disclosure of Connexin test results, the role of family history continued to be a significant factor for attributing a genetic explanation to deafness for participants who received Connexin-negative or Connexin-inconclusive results (FE *p*<0.0002, FE *p* = 0.03, respectively). Specifically, in the Connexin-negative group, 42.4% with deaf family members marked that their deafness is genetic in origin compared to 7.69% without deaf relatives; and in the Connexin-inconclusive group, 66.7% with deaf family members marked that their deafness is genetic compared to 12.5% without deaf relatives. In contrast, there was no association between family history and reason why deaf for the Connexin-positive group at 1-month post-test (FE *p* = 1.0) and>95.0% of these individuals marked that their deafness is genetic in origin. Results are nearly identical at 6-months post test result (Connexin-negative group FE *p* = 0.005; Connexin-inconclusive group FE *p* = 0.003; Connexin-positive group FE *p* = 0.14). Overall, these results provide evidence that participants have good understanding of their Connexin results, i.e., they understood their Connexin results in the context of the terms ‘Connexin-related deafness’ and ‘genetic,’ and in the context of their family history.

## Discussion

Deaf genetic testing raises a variety of ethical, social, and genetic counseling issues, yet there is a lack of empirical data on the actual impact of genetic counseling and genetic testing on deaf adults and the culturally Deaf community from which to address these issues. This article reports results from the only prospective, longitudinal study to address outcomes of deaf genetic testing and genetic counseling, in a sample of 209 deaf adults who underwent actual genetic counseling and Connexin-26 and Connexin-30 genetic testing and received their genetic test results. Our results provide insight into the effects of genetic counseling and testing on deaf individuals' identity and understanding of their genetic test results, for the period including pre- and post-test genetic counseling through six months after receipt of genetic test results. We found evidence that pre-test genetic counseling set appropriate expectations about test results and that participants understood their specific Connexin results following genetic counseling. However, we found no evidence that genetic information, provided in the context of linguistically and culturally appropriate genetic counseling, altered participants' deaf identity, despite having adequate power to detect moderate differences in identity change over time.

Deaf genetic testing raises questions about the psychological and social impact of genetic information on deaf individuals. In a recent article, we demonstrated that Connexin-26 and Connexin-30 test results can alter deaf adults' feelings of perceived personal control and anxiety, but that these results have no effect on feelings of depression [36]. Importantly, we found that deaf genetic testing can promote psychological well-being in deaf individuals who receive a positive Connexin result. In the current study we examined the effect of genetic counseling or Connexin-26 and Connexin-30 genetic testing on deaf identify using the DIDS-R subscales to measure four orientations of deaf identity: hearing, marginal, immersion, and bicultural. Consistent with other studies [12], [51], we found that language, age, family history, education level, and high school program were significantly associated with deaf identity, and hence are important for the formation of deaf identity. However, despite adequate power, we found no evidence that genetic counseling and Connexin genetic testing, or the nature of that result (e.g., Connexin-positive, Connexin-negative) meaningfully *changed* participants' deaf identity, at least out to 6-months post-test result. Furthermore, unsolicited comments from participants were consistent with the statistical results and provided some context for understanding these results. Because we found clear evidence that participants understood their genetic test results, the lack of impact of the Connexin result on deaf identity cannot be explained by “not understanding their results.” Our findings suggests that *knowing* the underlying reason why an individual is deaf is less relevant to the formation of deaf identity/cultural affiliation than socio-cultural and educational factors and that those individuals receiving positive genetic results are no more likely to change their identity/cultural affiliation than those individuals receiving negative genetic results.

In light of evidence that the relative frequency of Connexin-related deafness has increased in the non-Hispanic population in the United States in the last 100 years due to marriages between sign language users (linguistic homogamy), a language valued by the Deaf community, we hypothesized that Connexin-positive results would be enriched in our sample of non-Hispanic white culturally Deaf adults. Our results demonstrated that individuals self-reporting as affiliated with the Deaf community were significantly more likely to have Connexin-positive results than individuals reporting affiliation with the hearing community or with both Deaf and hearing communities. These results reveal the importance of deafness-causing variants in the Connexin-26 and Connexin-30 genes, albeit indirectly, to Deaf culture and the Deaf community in the United States, because these genetic variants are reflective of ethnic ancestry, family history, degree of deafness, age of onset, and language preference. It is interesting to note that participants indicated an interest in deaf genetic testing to strengthen the Deaf community [6]. [The survey item used lowercase d (…strengthen the deaf community…) but we use uppercase D here because analyses demonstrated that subjects who self-identified as affiliating with the Deaf community or with both Deaf and hearing communities more strongly endorsed this item than those self-identifying with the hearing community [6].] Our data suggest that the effects of Connexin-26 and Connexin-30 on strengthening the Deaf community will not come from changing deaf identity, but may come from the presence of deafness-causing variants in the population expanding the pool of hearing (children of Deaf adults) and deaf sign language users through the generations.

Deaf genetic testing is also complex for scientific reasons, and thus introduces complexity into genetic counseling for deafness. The scientific complexity arises because deafness is etiologically heterogeneous and because there are limits to our current ability to interpret some genetic test results. The current study underscores this complexity because 39.2% of participants received Connexin-positive results, 47.4% received Connexin-negative results, and 13.4% received Connexin-inconclusive results.

A Connexin-positive result reveals that the individual's deafness is genetic in origin, whereas, negative or inconclusive results do not provide definitive information about why an individual is deaf. Importantly, negative or inconclusive results do not rule out a genetic form of deafness because there are many untested genes that can explain why a person is deaf. Several studies of parents of a deaf child have documented that, in the absence of genetic counseling, this scientific complexity adversely affects parents' ability to understand inheritance of deafness, genetic information, and empiric recurrence chances when genetic testing or other clinical evaluation does not provide an explanation for why their child is deaf [39], [52], [53]. A common finding is that individuals tend to equate a negative genetic test result with ‘not genetic’ and a zero percent recurrence chance, reflecting the recognized phenomenon of misinterpretation of “residual chance” [54]. However, when genetic counseling is provided, parental understanding of their child's genetic test results is improved [45].

Deaf individuals' understanding of genetic test results may be additionally influenced by uneven access to information related to genetics concepts and genetics topics in ASL, and the use of sign language interpreters during encounters between deaf clients and non-ASL proficient genetics professionals. In a previous analysis of this study sample, we demonstrated that deaf adults' general knowledge and understanding of genetics concepts, heterogeneity of deafness, and genetic testing are enhanced by pre-test genetic counseling provided in a culturally and linguistically sensitive manner [40].

Here we demonstrate other outcomes of the effectiveness of genetic counseling because we found in this sample of deaf adults that individuals who received positive test results felt this was a more likely outcome of genetic testing immediately following the pre-test genetic counseling session than those who did not receive positive results. Furthermore, we found at 1- and 6-months after result disclosure that those who received positive test results understood that they have Connexin-related deafness and that this is genetic in origin even if they do not have any deaf relatives, which suggests that through genetic counseling one aspect of the meaning of positive test results was successfully conveyed. Moreover, individuals who received an inconclusive test result accurately reported that the reason they are deaf is undetermined, that it is still somewhat likely that they have Connexin-related deafness, and that the presence of deaf relatives is a meaningful predictor of having a genetic type of deafness. Finally, participants who received negative test results accurately reported that it was unlikely that they have Connexin-related deafness, that the reason they are deaf is undetermined, and that the presence of deaf relatives is a meaningful predictor of having a genetic type of deafness. Similar results were observed in a sample of hearing parents of a deaf child following genetic counseling [45], suggesting that the knowledge outcomes of genetic counseling for deaf genetic testing are generalizable across deaf and hearing clients.

This study has several strengths, including the high response rate at each assessment time point, and the lack of evidence for response bias between questionnaire responders and non-responders. However, there are several limitations that warrant discussion. First, the individuals who chose to participate in this study may differ in important ways from those who did not participate. As one example, our study participants were more highly educated and had a higher median income than a national sample of prelingually deaf adults [55]. Second, ∼8% of individuals determined eligible to participate in the study ceased participation either prior to submitting a DNA sample or prior to receiving results. These individuals were more likely to be male, more likely to be ASL-users, less likely to self-identify as white, and more strongly endorsed the DIDS-R immersion subscale items than those who continued through the protocol to receive their genetic test results. These factors raise questions about whether these participants were uncomfortable with lack of direct communication with the researchers, a non-ethnically diverse research team, or potential medicalization of deafness. Hence, generalizing our findings to the general deaf population should be done with caution. However, because the majority of this group was enrolled in the final six months of the study compared to those who continued to receive their genetic test results, it is possible that the most relevant factor for discontinued participation was simply related to timing of participation.

Another important limitation is that the average age of the sample was 45.9 years at enrollment, and baseline assessment of the deaf identity subscales indicated that most of the participants endorsed the bicultural identity and rejected the other three identities. Deaf identity is conceptualized as a construct that develops over time culminating in the bicultural identity, and since our sample was composed of adults we may have been less likely to observe changes to deaf identity due to genetic test results. It would be important to assess the effect of deaf genetic testing on the development of deaf identity in younger deaf individuals. In addition, this study evaluated whether participants' view of their own deaf identity changed, but it would also be interesting to study if others within the Deaf community view a persons' identity differently if they are found to have Connexin-positive result. Finally, this study did not specifically measure participants' views about a connection between Deaf culture and the Connexin-related genes. Thus, one potential explanation for our results is that our participants were not aware of this potential connection. If this connection was widely known in the Deaf community then there is a chance that learning Connexin results would change deaf identity, and this is a topic for future research.

## Conclusions

In summary, this is a prospective, longitudinal study to determine the impact of genetic counseling or genetic testing on deaf identity and understanding of genetic test results. To date, we have demonstrated that some deaf individuals are interested in deaf genetic testing [6], that pre-test genetic counseling enhances participants' general knowledge outcomes regarding genetics concepts, heterogeneity of deafness, and genetic testing [40]; and that individuals who receive a Connexin-positive result experience psychological well-being [36]. This study demonstrates that pre-test genetic counseling appropriately sets expectations about Connexin results; that post-test genetic counseling enhances participants' understanding of their specific Connexin results; that deaf identity is not changed as a function of genetic counseling or learning a Connexin result; and that the formation of or change in deaf identity is not directly related to Connexin results, but it is based on other variables such as age, language, family history, education level, and high school program. We hypothesize that deafness-causing variants in Connexin-26 and Connexin-30 play an important indirect role on the Deaf community.

## Supporting Information

Table S1 **Repeated measures regression analyses: Least square estimates and standard errors for main effects, interaction term, and covariates.**
(DOCX)
