# Supplementary material for: Impact of Genetic Counseling and Connexin-26 and Connexin-30 Testing on Deaf Identity and Comprehension of Genetic Test Results in a Sample of Deaf Adults: A Prospective, Longitudinal Study
Source: PLoS One. 2014 Nov 6;9(11):e111512. doi: 10.1371/journal.pone.0111512 (PMC4222828; doi:10.1371/journal.pone.0111512)
Supplement: Table S1 — Repeated measures regression analyses: Least square estimates and standard errors for main effects, interaction term, and covariates. (DOCX) [file pone.0111512.s001.docx]

Table S1. Repeated measures regression analyses: Least square estimates and standard errors for main effects, interaction term, and covariates.

| Predictor variable | Response category | Deaf Identity Development Scale-Revised, Subscale | | | | | | | | Perceived Chance | |
| --- | --- | --- | --- | --- | --- | --- | --- | --- | --- | --- | --- |
|  |  | Hearing | | Marginal | | Immersion | | Bicultural | |  | |
|  |  | Estimate | SE | Estimate | SE | Estimate | SE | Estimate | SE | Estimate | SE |
| Connexin^a^ | Neg | 3.95 | 0.07 | 3.89 | 0.07 | 3.43 | 0.06 | 2.00 | 0.05 | 1.79* | 0.06 |
|  | Inc | 3.92 | 0.11 | 3.92 | 0.06 | 3.54 | 0.10 | 2.04 | 0.09 | 2.11 | 0.10 |
|  | Pos | 3.99 | 0.08 | 4.01 | 0.08 | 3.56 | 0.07 | 2.02 | 0.06 | 2.95 | 0.07 |
| Time | Baseline | 3.99 | 0.06 | 3.93 | 0.06 | 3.48 | 0.06 | 2.03 | 0.05 | 2.17* | 0.07 |
|  | Pre-Test | 3.94 | 0.06 | 3.94 | 0.06 | 3.50 | 0.06 | 1.98 | 0.05 | 2.21 | 0.07 |
|  | 1-M Post-Test | 3.97 | 0.06 | 3.96 | 0.06 | 3.54 | 0.06 | 2.03 | 0.05 | 2.37 | 0.07 |
|  | 6-M Post-Test | 3.91 | 0.06 | 3.92 | 0.06 | 3.52 | 0.06 | 2.04 | 0.05 | 2.38 | 0.08 |
| Age | <45 years | 4.07* | 0.07 | 3.99 | 0.07 | 3.54 | 0.06 | 2.03 | 0.05 | 2.30 | 0.06 |
|  | >=45 years | 3.84 | 0.07 | 3.89 | 0.08 | 3.48 | 0.07 | 2.01 | 0.06 | 2.27 | 0.06 |
| Education | <bachelor degree | 3.73* | 0.07 | 3.68* | 0.08 | 3.48 | 0.07 | 2.04 | 0.06 | 2.30 | 0.06 |
|  | >=bachelor degree | 4.17 | 0.07 | 4.19 | 0.07 | 3.53 | 0.06 | 2.00 | 0.05 | 2.26 | 0.06 |
| Language | ASL | 4.16* | 0.05 | 3.97 | 0.06 | 3.07* | 0.05 | 1.86* | 0.04 | 2.28 | 0.05 |
|  | ASL+English | 4.10 | 0.08 | 3.93 | 0.09 | 3.39 | 0.08 | 1.76 | 0.07 | 2.21 | 0.08 |
|  | English | 3.60 | 0.12 | 3.91 | 0.13 | 4.07 | 0.11 | 2.44 | 0.10 | 2.36 | 0.11 |
| Deaf relatives | No | 3.85* | 0.07 | 3.79* | 0.07 | 3.49 | 0.07 | 2.06 | 0.06 | 2.08* | 0.06 |
|  | Yes | 4.06 | 0.07 | 4.08 | 0.07 | 3.53 | 0.07 | 1.98 | 0.06 | 2.48 | 0.06 |
| High School program^b^ | Hearing | 3.89 | 0.07 | 3.83 | 0.08 | 3.68* | 0.07 | 2.13 | 0.06 | 2.16* | 0.06 |
|  | Deaf | 4.10 | 0.09 | 4.09 | 0.09 | 3.33 | 0.08 | 2.01 | 0.07 | 2.49 | 0.08 |
|  | Mainstream | 3.88 | 0.09 | 3.83 | 0.07 | 3.49 | 0.08 | 1.96 | 0.07 | 2.19 | 0.08 |
|  | Mixed | 3.94 | 0.12 | 3.99 | 0.13 | 3.54 | 0.12 | 1.99 | 0.10 | 2.29 | 0.11 |
| Time x Connexin | Baseline:Neg | 3.98* | 0.07 | 3.88 | 0.07 | 3.43 | 0.07 | 2.05 | 0.06 | 2.09* | 0.08 |
|  | Baseline:Inc | 4.01 | 0.11 | 3.89 | 0.12 | 3.46 | 0.11 | 2.05 | 0.10 | 2.21 | 0.15 |
|  | Baseline:Pos | 3.98 | 0.08 | 4.00 | 0.08 | 3.55 | 0.08 | 2.00 | 0.07 | 2.21 | 0.09 |
|  | Pre-Test:Neg | 3.92 | 0.07 | 3.91 | 0.07 | 3.40 | 0.07 | 1.93 | 0.06 | 2.07 | 0.08 |
|  | Pre-Test:Inc | 3.89 | 0.11 | 3.90 | 0.12 | 3.50 | 0.11 | 2.05 | 0.10 | 2.05 | 0.14 |
|  | Pre-Test:Pos | 4.01 | 0.08 | 4.02 | 0.08 | 3.60 | 0.08 | 1.97 | 0.07 | 2.52 | 0.09 |
|  | 1-M Post-Test:Neg | 3.92 | 0.07 | 3.85 | 0.07 | 3.44 | 0.07 | 2.00 | 0.06 | 1.48 | 0.09 |
|  | 1-M Post-Test:Inc | 3.98 | 0.12 | 4.02 | 0.12 | 3.63 | 0.12 | 2.01 | 0.10 | 2.12 | 0.15 |
|  | 1-M Post-Test:Pos | 3.99 | 0.08 | 4.02 | 0.09 | 3.55 | 0.08 | 2.06 | 0.07 | 3.52 | 0.10 |
|  | 6-M Post-Test:Neg | 3.98 | 0.07 | 3.92 | 0.07 | 3.46 | 0.07 | 2.01 | 0.06 | 1.51 | 0.09 |
|  | 6-M Post-Test:Inc | 3.80 | 0.12 | 3.85 | 0.12 | 3.56 | 0.11 | 2.06 | 0.10 | 2.08 | 0.16 |
|  | 6-M Post-Test:Pos | 3.96 | 0.08 | 3.99 | 0.09 | 3.53 | 0.08 | 2.05 | 0.07 | 3.54 | 0.10 |

* variable is a significant predictor of outcome variable at p<0.05

*Note*. ^a^ Neg= negative Connexin result, Inc = inconclusive Connexin result, Pos = positive Connexin result; ^b^ As in Boudreault et al 2010, deaf-based high school indicates predominantly ASL or coded communication in the classroom; hearing-based high school indicates predominantly oral instruction in the classroom without interpreter/support services; mainstream high school indicates public school that predominantly provides sign instruction with interpreter/support services; and mixed indicates attending two or more of the previously described high school programs
